# Sensory Profiling of Burdekin Plum Leathers and Consumer Acceptability of Its Combination With Trail Mix

**DOI:** 10.1002/fsn3.70277

**Published:** 2025-05-12

**Authors:** Gengning Chen, Sandra Milena Olarte Mantilla, Michael E. Netzel, Daniel Cozzolino, Yasmina Sultanbawa

**Affiliations:** ^1^ ARC Industrial Transformation Training Centre for Uniquely Australian Foods, Queensland Alliance for Agriculture and Food Innovation The University of Queensland Indooroopilly Queensland Australia

## Abstract

*Pleiogynium timoriense* is commonly referred to as Burdekin plums (BPs) in Australia. Despite BP fruits being traditionally consumed by Indigenous Australians, little research on their potential food applications is available. With the increasing demand for healthy snacks and the importance of sensory quality in consumer choice of food products, this study explored the sensory profiles and acceptability of five formulations of BP fruit leathers (20BP, 40BP, 60BP, 80BP, and 100BP, containing 20–100% BP). Sensory profiling of the five BP fruit leathers revealed that most attributes were correlated. Both 80BP and 100BP were similar and characterized by significantly (*p* < 0.05) higher *dark fruit flavor*, *astringency,* and *firmness*, while 20BP fruit leather was significantly (*p* < 0.05) higher in *cooked yellow fruit flavor* and *dissolving texture.* The acceptability taste test showed that 20BP, 40BP, and 60BP were the preferred formulations. 40BP was selected for further consumer study. The acceptability of 40BP when consumed with a commercial fruit and nut trail mix (TM) was studied. Then, 121 consumers rated the acceptability of three samples: 40BP, TMBP (40BP mixed with TM), and TM. The results showed that all three samples were liked. Moreover, the sensory acceptability and purchase intention of TMBP were comparable to TM, which was higher than 40BP alone. Furthermore, cluster analysis revealed that the cluster with higher liking scores consumed TM more often and had a higher willingness to consume Australian native fruits. The study showed the promising food applications and acceptability of BP leathers and their incorporation in a commercial TM.

## Graphical abstract

Sensory profiling of Burdekin plum fruit leathers showed most attributes were correlated. Burdekin plum fruit leathers and its addition in trail mix were well received by consumers. Adding Burdekin plum fruit leather to trail mix can increase the acceptability of fruit leather. Consumer familiarity with food and attitudes to Australian native fruits affected liking scores.

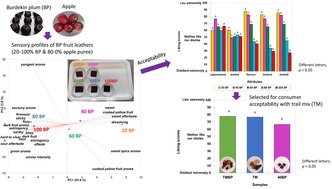

## 1 Introduction

Snacking between main meals has become more popular in recent years due to changes in life and work styles. Popular snacks such as sweets, potato chips, and soft drinks are ultra‐processed and high in sugar, salt, and saturated fat (Machado et al. 2019; Piernas and Popkin 2010) and can contribute to the development of obesity, diabetes, and cardiovascular diseases if consumed excessively (Almoraie et al. 2021; Dunford and Popkin 2018). Consumers are becoming more conscious about snacks that are not only tasty, convenient, and low priced, but also rich in healthy ingredients (Potter et al. 2018). Fruits are one of the healthy snack alternatives that have been commonly promoted and recommended to the general public (Potter et al. 2018). Consuming fruits instead of unhealthy and ultra‐processed snacks has been linked to improved physical and mental well‐being (Smith and Rogers 2014). The health benefits associated with fruit consumption are well documented and linked to the favorable nutritional composition of fruits (low in energy, high in fiber, micronutrients, and phytochemicals) (Cione et al. 2020).

The major barriers for increasing the consumption of fruits as healthy snack alternatives are costs, convenience, and taste preferences when compared to their ultra‐processed counterparts (Harris et al. 2023). Dried fruit products can be an option to boost fruit consumption because they are often considered more convenient as they are lighter in weight and have a longer shelf life, which facilitates convenient storage, transportation, and enables consumers to enjoy fruits beyond their typical seasons (Testa et al. 2023). In terms of taste, dried fruit products usually have enhanced flavor intensity compared to fresh fruits (Petikirige et al. 2022). Fruit leathers, also referred to as fruit bars or roll‐ups, have been a popular dried fruit snack in North America and Asia, and have been branded toward the gourmet healthy foods sector in recent years (Torres et al. 2015).

*Pleiogynium timoriense* (DC.) Leenh, an Australian native plant, produces plum‐like fruits, commonly referred to as Burdekin plums (BPs) (Low 1991). The fruits are nutritious and have strong antioxidant capacity, being traditionally consumed by Indigenous people (Chen et al. 2023). Despite the pleasant overall flavor of the fruits, the sour and astringent characters in the flavor profile and the thin layer of flesh and a large stone diminish the eating quality and make the fruit less competitive against other fruits such as mangoes, one of its close relatives (Rozefelds and Kane 2016) or cultivated plums (Low 1991).

However, taste has often been identified as the most important determinant of food choice despite the increasing focus on health (Aggarwal et al. 2016; Forbes et al. 2016). Sensory perception has been known to differ when food is consumed alone compared to when it is consumed together with other foods (van Eck and Stieger 2020). This change in sensory perception has been utilized to develop novel food products by incorporating non‐mainstream foods and ingredients into mainstream foods, creating new and appealing flavor profiles to attract consumers (Traynor et al. 2021). Fruit leathers can be made by blending different fruits together to produce the desired quality without adding additives (da Silva Simão et al. 2020). For example, guava has been incorporated into papaya leather to reduce the unpleasant papaya odor and enhance the overall sensory quality (Kumar et al. 2008). Apples are one of the most popular fruits in making fruit leathers and have been used in blended fruit leathers to create the desired quality such as increased sweetness and reduced tartness (Torres et al. 2015). By removing the large stone and blending the BP puree with apple puree, BP fruit leathers with different BP to apple ratios were produced (Chen et al. 2024). However, their sensory characteristics and consumer acceptability have not been studied.

Fruit leather can be consumed without or with other foods such as ice cream, nuts, and breakfast cereals (Irwandi et al. 1998). Studies have found that dried fruits in combination with other food products such as breakfast cereals tend to be more frequently consumed than dried fruits alone (Jesionskowska et al. 2008). Nuts and trail mix (TM) are among the fastest‐growing snack categories in the United States (Forbes et al. 2016). Overall, fruits and nuts are highly recommended as snacks in many countries around the world (Potter et al. 2018). Pairing nuts and dried fruit in the diet can reduce the glycemic response (Zhu et al. 2018) and the risk of cardiovascular disease (Carughi et al. 2016). The combination of lesser‐known food products such as BP leather with mainstream foods could be an effective marketing strategy to increase their popularity (Carvalho et al. 2011; Papies et al. 2020; Possidónio et al. 2021) and also a point of differentiation to already available mainstream products (Rabadán et al. 2021).

To the best of our knowledge, there is little research published about the sensory descriptive analysis of blended fruit leather and the acceptability of the fruit leather consumed alone or together with other foods. One study investigated the appearance, taste, texture, and overall acceptability of fruit leathers made from a mixture of banana, pineapple, and apple, before and after their incorporation into cakes by 20 panelists. The results showed that the cakes containing fruit leather had comparable acceptability scores to cakes containing a market‐fresh fruit mix and to the fruit leathers served on their own (Offia‐olua and Ekwunife 2015). This study aimed to explore the sensory characteristics of BP fruit leathers with different levels of apple addition and the acceptability when consumed together with a commercial TM to address the increasing demand by consumers for healthy snacks.

## 2 Materials and Methods

### 2.1 Sensory Profiling of Five BP Leathers

**FIGURE 1:**
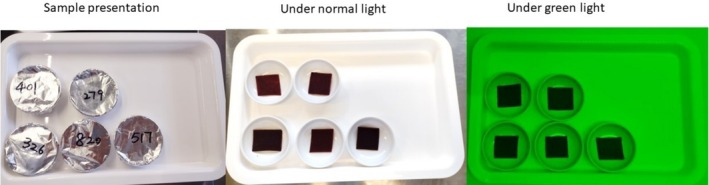
BP leather samples presented to panelists with a three‐digit random code, as well as under normal and green lights.

Five formulations of BP leathers prepared by mixing BP with apple puree in different ratios: 100BP (100% BP), 80BP (80% BP and 20% apple puree), 60BP (60% BP and 40% apple puree), 40BP (40% BP and 60% apple puree), and 20BP (20% BP and 80% apple puree). Further details are described in our previous work (Chen et al. 2024). The conventional descriptive sensory analysis method was used to profile the aroma, flavor, texture, and aftertaste of the five fruit leather formulations. A benchtop tasting with 10 experienced tasters was conducted to generate relevant lexicons and to develop appropriate sample presentation conditions, including the serving quantity, palate cleanser, and lighting. The serving size of 4 cm^2^ of fruit leather (approx. 1 g) was enough to conduct the sensory profiling. Color differences were noticed between the different fruit leather formulations and therefore, it was decided to use green light in the sensory booths to mask the color effect during the formal sensory profiling session (Figure 1).

Twelve trained panelists, eleven females and one male, aged between 21 and 68 years, were selected from a pool of experienced sensory panelists who had been assessed for sensory acuity testing previously. The evaluation was conducted in the sensory laboratory (Long Pocket Campus, University of Queensland, Indooroopilly, QLD, Australia). The study was approved by the UQ Human Ethics Committee (code: 2019002607) and informed consent from panelists was obtained before the evaluation.

Four training sessions (one hour each) were conducted to generate a lexicon, consensus of definitions, reference standards, and assessment methods as well as to train the panelists in using the sensory scales. A total of 26 attributes were generated and consolidated into 23 attributes, as shown in Table 1. One formal session with triplicate presentation of samples to panelists (totaling 15 samples) was held on the same day. Each sample was placed in a plastic disk covered with aluminum foil and labeled with a three‐digit random code. Samples were presented in sets of five arrangements using a randomized complete block design. There was a 90 s break between each sample and a 10 min break between each replicate to minimize sensory fatigue. Line scales with anchors none/low (0) to high (100) were used, except for the attributes including firmness (0 for soft, 100 for hard), dissolving (0 for hard to dissolve, 100 for easy to dissolve) and hard to clear (0 for easy to clear, 100 for difficult to clear).

**TABLE 1:** Sensory vocabulary, definitions and reference standards agreed by the panelists.

Attribute	Definition	Reference standard
Aroma		
Aroma intensity	The overall aroma intensity of the sample	Nil
Dark fruit	The aroma of fresh and/or stewed dark fruit like blackberry, plum, red currant, rhubarb, and tamarind (none to high)	¼ tsp. cooked black berries (Coles Frozen Fruit Blueberries, Hawthorn, VIC, Australia) + ¼ tsp. cooked queen garnet plum + ¼ tsp. cooked rhubarb
Cooked yellow fruit	The aroma of fresh and/or stewed, cooked yellow fruits like apple, pear, and apricot (none to high)	½ tsp. cooked red gala apple +1 peace diced pear in juice (SPC, Shepparton, VIC, Australia) + ¼ tsp. spoon dry apricot (Woolworths brand, Bella Vista, NSW, Australia) + 1 drop apple juice (Sunraysia, Melbourne, VIC, Australia)
Green	A fresh or dried green grassy or dry note (none to high)	¼ tsp. fresh cut grass tsp. + ¼ tsp. dried oregano (Hoyt's Food, Moorabbin, Victoria, Australia)
Savory	A savory note, like ham, bacon, fishy, seaweed (none to high)	¼ tsp. leg ham (Champagne, D'Orsogna Limited, Palmyra, WA, Australia), ½ tsp. smoked bacon (Shortcut bacon, Woolworth, Bella Vista, NSW, Australia), ½ flake bonito flakes (Yamaki, Jun Pacific, Chatswood, NSW, Australia), ¼ tsp. nori seaweed (Obento, Oriental Merchant, Laverton North, VIC, Australia)
Sweet spice	A sweet spice note like cloves, cinnamon (none to high)	One pinch ground cloves (Hoyt's Food, Moorabbin, Victoria, Australia) + 1 pinch cinnamon powder (Hoyt's Food, Moorabbin, Victoria, Australia)
Pungent	The vinegary, sweaty, gluey and metallic pungent aroma intensity (none to high)	One drop brown vinegar (Woolworths homebrand, Bella Vista, NSW, Australia) + ¼ tsp. pva glue (Craft Smart, CSA trading, Notting Hill, VIC, Australia)
Flavor		
Sour	The sour, tangy, zesty flavor reminding of lemon or sherbet (low to high)	½ tsp. fresh lemon
Sweet	The sweetness of the sample (low to high)	Nil
Dark fruit	The flavor of fresh and/or stewed dark fruit like blackberry, plum, red currant, rhubarb, and tamarind (none to high)	As per aroma
Cooked yellow fruit	The flavor of fresh and/or stewed, cooked yellow fruits like apple, pear, and apricot (none to high)	As per aroma
Earthy	The earthy, metallic, black tea flavor (none to high)	One tsp. fresh forest soil + ¼ tsp. black tea (Twinings traditional afternoon, AB food and beverages, Rowville, VIC, Australia)
Texture		
Firmness	The firmness of the sample, from low being soft, tender, and almost papery to high being leathery and hard	Nil
Dissolving	The sensation of the main bolus becoming smaller and dissolving away almost melting. From low being hard to dissolve to high being easier to dissolve	Nil
Bitsy	The perception of grainy and/or fibrous particles in the mouth from low having less bitsy particles to high having more bitsy particles	Nil
Sticky	The sticky sensation perceived, almost adhering, between the top and bottom molar cusps while chewing (low to high)	Nil
Fizzy	The fizzy and sherbet feeling of the sample while chewing (none to high)	One tsp. sherbet (wizz fizz, FYNA foods, Hallam, VIC, Australia)
Astringency	The astringency perceived from the sample while chewing (low to high)	½ tsp. natural Greek yogurt (Chobani Plain Greek yogurt, Dandenong South, VIC, Australia)
Taste and mouthfeel after swallowing		
Sour	The sour taste perceived from the sample after swallowing (low to high)	Nil
Sweet	The remaining sweetness perceived from the sample after swallowing (low to high)	Nil
Fruit length	The fruit flavor lingering intensity remaining in the mouth after swallowing (low to high)	Nil
Astringency	The astringent and drying sensation perceived in the mouth surfaces after swallowing the sample	As per above
Hard to clear	The easiness to clear the pieces of the samples from the mouth from easy being low to high being hard	Nil

### 2.2 Preliminary Acceptability of the Five BP Leathers

Following the formal descriptive evaluation, the same panelists were asked to rate the acceptability of the fruit leathers. The appearance, aroma, flavor, texture, and overall liking were rated on a scale (0–100) anchored at both ends and the middle from dislike extremely to neither like nor dislike to like extremely (Embling et al. 2022), using the Redjade Sensory Software (Pleasant Hill, CA, USA). For each attribute, panelists were also asked to describe what they liked or disliked about the sample. The presentation of samples was the same as described above, except samples were served under normal light conditions. One BP leather formulation was selected for further acceptability tests with TM and proximate analysis.

### 2.3 Acceptability of Selected BP Leather and TM

The acceptability test took place at TropAg 2022, an international tropical agriculture conference hosted by Queensland Alliance for Agriculture and Food Innovation and the University of Queensland in Brisbane. More than 1000 people from 53 countries studying or working in food and agriculture‐related fields attended this conference. Conference attendees were invited to participate in the acceptability tests, and 121 participants completed the test. Each participant was briefed about the study and testing design and signed a consent form before the evaluation.

Participants evaluated three samples, including 40BP, commercial fruit and nut TM and the combination of TM and 40BP (TMBP). The composition of the samples is shown in Table 2. The samples were stored in individual polypropylene containers with three‐digit codes and presented in sets of three arrangements using a randomized complete block design. Consumers were asked to rate each sample for overall liking on a labeled affective magnitude scale (Schutz and Cardello 2001) and to indicate their purchasing intention. Water was used as a palate cleanser between the samples. Demographic information was collected at the end of the questionnaire, as well as information about the frequency of purchasing fruit leather and TM and the willingness to eat native fruits. Data were collected on tablets using the Redjade Sensory Software.

**TABLE 2:** Samples for the acceptability evaluation.

Sample		Fruit and nut trail mix (TM)	40BP	Trail mix with 40BP (TMBP)
Sample image		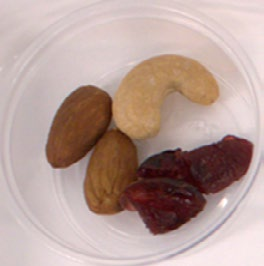	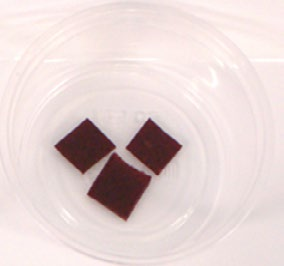	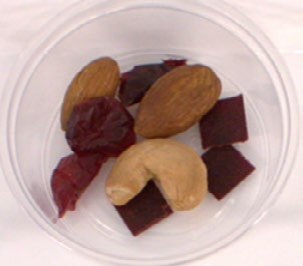
Sample code		531	687	924
Ingredients (g/serve)	Roasted almonds	3.9	0	3.9
	Roasted cashews	1.7	0	1.7
	Cranberries (cranberries, sugar, sunflower oil).	4.2	0	4.2
	40BP	0	1	1

### 2.4 Proximate Composition

Samples were analyzed for water content (AOAC 925.09), crude fat (960.39), protein (990.03), total ash (923.03), and dietary fiber (985.29) (AOAC 2000). The available carbohydrate content was calculated by subtracting water, protein, fat, ash, and dietary fiber from 100% (AOAC 2000).

### 2.5 Data Analysis

Data analysis was done using XLSTAT (Addinsoft 2022, Paris, France). For the descriptive panel evaluation, a mixed ANOVA model was selected with samples as fixed effect and panelists, sessions, and panelists × sessions interactions as random effects. A principal component analysis (PCA) was conducted on the mean attribute scores to explore sample association. Another PCA was conducted for the mean attribute scores with physicochemical data of fruit leathers from previous work (Chen et al. 2024) as supplementary data to explore the association of sensory attributes and physicochemical parameters.

For consumer acceptability evaluation, agglomerative hierarchical clustering (AHC) based on the Ward's method was used to classify consumer clusters based on liking data. ANOVA was applied to test the differences between samples. Tukey–Kramer HSD was conducted following ANOVA for pairwise comparison (*p* < 0.05). Chi‐square analysis followed by the Marascuilo procedure was used to test the difference in purchase intention as well as associations between clusters and consumer traits.

## 3 Results and Discussion

### 3.1 Sensory Profiling of Five BP Leathers

#### 3.1.1 Evaluation of Panel Performance

Panelist's performance scores were checked for discrimination power across samples and repeatability among replicates before analyzing and interpreting results to ensure the robustness of the data (Table S1). In terms of discrimination power, 11 out of the 12 panelists differentiated at least 12 out of 23 attributes. For repeatability, 11 out of 12 panelists were able to give repeatable results for 14 attributes. One panelist had relatively low repeatability results compared to the other panelists. After removing this panelist, the dark fruit aroma attribute, which is of interest for these samples, was significantly (*p* < 0.05) different. Therefore, the results of this panelist were removed from further descriptive sensory analysis. The degree of scale used by 11 panelists and the sample diversity were evaluated by calculating the mean, standard deviation, coefficient of variation, minimum and maximum of each attribute rated by each panelist (Table S2) (Grace et al. 2020). The effects of samples, panelists and replicates on the attribute scores are shown in Table S3. Four attributes, green aroma, savory aroma, sweet spice aroma and fruit length had low contributions to the discrimination. However, 19 out of 23 attributes had discriminating power among the tested samples. In terms of the panel's effect on attributes, most attributes except dissolving had a significant difference, indicating that panelists used the scales differently, which is typical in conventional descriptive analysis (Sinesio et al. 2010). There was no significant (*p* > 0.05) effect of replicates on attributes, except green aroma and dark fruit, indicating the result is reproducible and the training was adequate. Overall, the scale was well used as the minimum and maximum covered both sides of the scale, and the variability of samples was displayed for most attributes. Therefore, the performance of 11 panelists and their ratings for 23 attributes were considered for further evaluation.

#### 3.1.2 Sensory Profiles of BP Leathers

**FIGURE 2:**
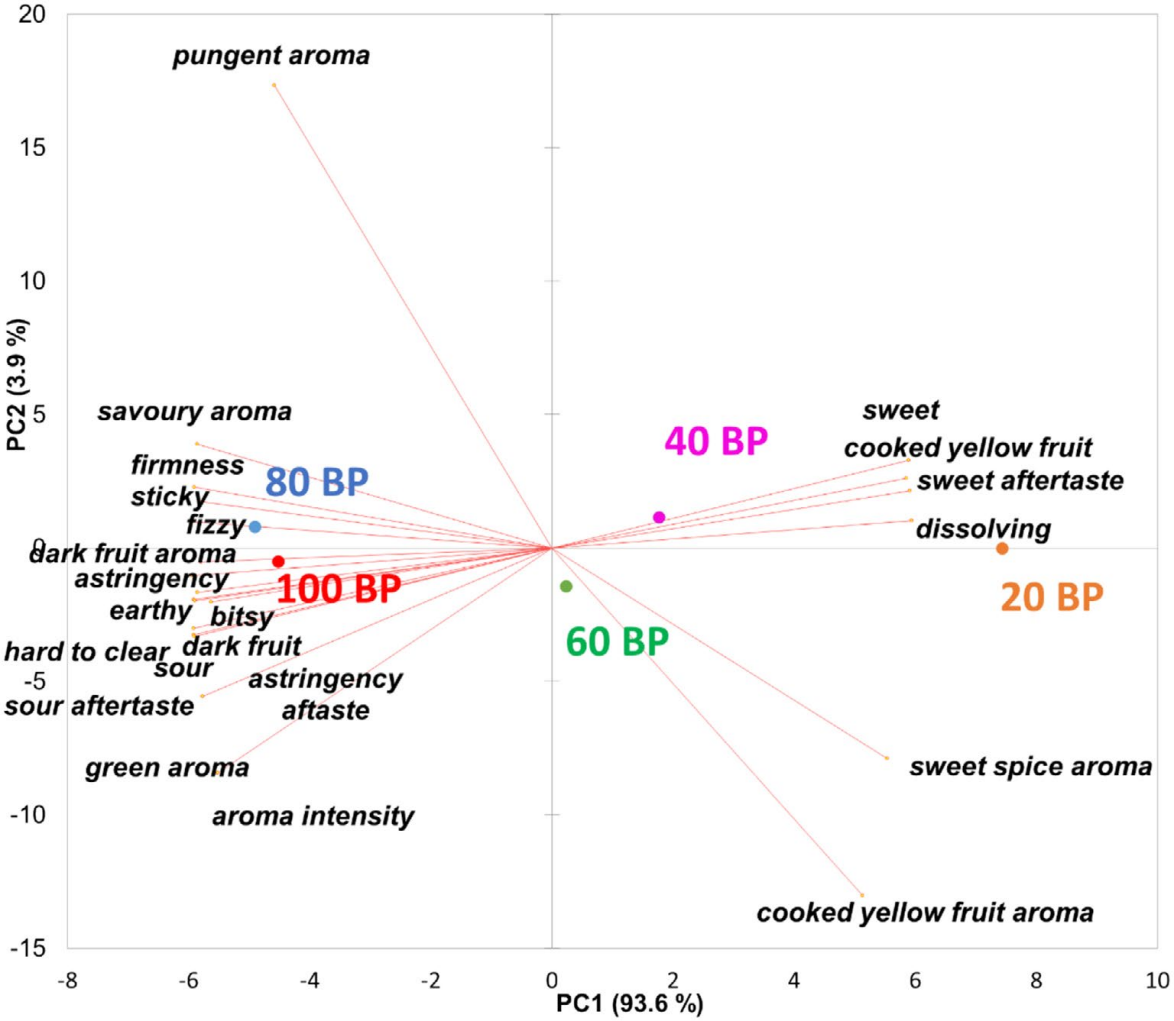
PCA biplots of the sensory properties of five formulations of BP leathers (*n* = 11 panelists × three replicates).

The sensory scores of five formulations of BP leathers are shown in Table 3. To highlight the difference among samples, a PCA was conducted. The PCA biplot with 22 attributes (except *fruit length*) and 5 samples is shown in Figure 2. The first two principal components explained 97.5% of the total variation in the dataset. The first component (PC1) accounted for 93.6% of the total variation with heavy loadings for most attributes. The second component (PC2) accounted for only 3.9% with mainly aroma components including *pungent, cooked yellow fruit, sweet spice*, and *aroma intensity* being the major contributors. This is an indication that most attributes were highly correlated. *Sweet taste* and *aftertaste*, *cooked yellow fruit taste*, and *dissolving texture* were positively correlated with each other, but negatively correlated with *dark fruit taste* and *aroma, sour taste* and *aftertaste, astringency taste* and *aftertaste, savory aroma, green aroma, earthy, bitsy, firmness, sticky, fizzy*, and *aroma intensity*. Furthermore, *sweet spice* and *cooked yellow fruit aroma* were positively correlated, but negatively correlated with a *pungent aroma*. Sample 20BP had the highest loading of attributes positively correlated with sweet, while 80BP and 100BP had the lowest loadings. The plot also showed a clear separation of 20BP from 80BP and 100BP on PC1, and a clear clustering of 100BP and 80BP. Both 40BP and 60BP exhibited intermediate behavior with a certain degree of separation. This corresponded to the sensory scores shown in Table 3. Sample 100BP was similar to that of 80BP in the tested sensory attributes (ANOVA, *p* < 0.05). While 20BP had lower scores in the following 14 attributes: *aroma intensity, pungent aroma, dark fruit aroma* and *taste, sour taste* and *aftertaste, earthy taste, firmness, bitsy, sticky, fizzy, astringency taste and aftertaste*, and *hard to clear*, the scores for *cooked yellow fruit aroma* and *taste, sweet taste* and *aftertaste*, and *dissolving* were higher. Furthermore, the scores of 40BP and 60BP leathers were between 20BP and 100BP. Both 40BP and 60BP were similar in most attributes except for *bitsy, astringency aftertaste, sour taste* and *aftertaste* (lower scores for 40BP) as well as *cooked fruit taste, sweet taste*, *and aftertaste* (higher scores for 40BP). Overall, the addition of 20% apple puree did not affect (*p* > 0.05) the sensory attributes of the BP leathers, whereas the addition of 80% apple puree had a significant (*p* < 0.05) effect on 19 attributes.

**TABLE 3:** Sensory attribute mean scores of five formulations of BP leathers (*n* = 11 panelists × three replicates).

Sensory attribute	100 BP	80 BP	60 BP	40 BP	20 BP	*p*
Aroma (none–high, 0–100)						
Aroma intensity	64.0a	64.9a	59.6ab	49.3b	47.4b	0.002
Dark fruit	58.0a	58.0a	53.8ab	52.2ab	41.4b	0.045
Cooked yellow fruit	24.8 24.8b	24.0b	35.2ab	27.0b	40.2a	0.013
Green	34.4a	33.8a	30.8a	27.7a	25.8a	0.490
Savory	30.5a	31.0a	25.1a	25.4a	20.9a	0.366
Sweet spice	13.8a	10.8a	15.6a	15.3a	18.8a	0.586
Pungent	33.2ab	44.6a	19.2c	30.4bc	19.6c	0.001
Flavor (none–high, 0–100)						
Sour	82.4a	81.2a	59.0b	42.5c	12.1d	< 0.001
Sweet	8.5d	14.0d	39.1c	55.9b	82.8a	< 0.001
Dark fruit	69.6a	77.0a	68.1ab	58.0b	32.1c	< 0.001
Cooked yellow fruit	15.9d	19.4d	34.2c	45.4b	81.4a	< 0.001
Earthy	48.0a	43.2a	27.0b	21.5b	8.1c	< 0.001
Texture (none–high, 0–100)						
Firmness	87.8a	87.4a	45.8b	44.5b	7.4c	< 0.001
Dissolving	14.4c	16.5c	44.1b	54.5b	90.2a	< 0.001
Bitsy	77.2a	79.9a	54.3b	40.1c	17.8d	< 0.001
Sticky	49.3a	50.8a	42.6a	41.9a	25.4b	0.015
Fizzy	67.8a	73.6a	47.4b	40.9b	11.5c	< 0.001
Astringency	78.1a	78.0a	53.8b	43.9b	14.2c	< 0.001
Taste and mouthfeel after swallowing (none–high, 0–100)						
Sour	74.5a	72.5a	52.4b	37.2c	11.3d	< 0.001
Sweet	8.9d	12.3d	36.1c	47.9b	71.6a	< 0.001
Fruit length	53.4a	60.6a	53.4a	52.7a	58.8a	0.791
Astringency	72.5a	69.4a	50.0b	35.8c	11.7d	< 0.001
Hard to clear	71.3a	65.6a	44.4b	35.6b	14.8c	< 0.001

*Note:* Different letters within each row indicate significant differences between samples (Tukey HSD at *p* < 0.05).

The proximity of 80BP and 100BP could be either attributed to the saturation of sour taste (Curtis et al. 1984) or the masking of sweetness by sourness at a high acid concentration (Pangborn 1961). Interestingly, 80BP had slightly higher scores in dark fruit taste and fizzy texture than 100BP. Similar effects were reported in mango, where the addition of sugar enhanced the perception of different flavor attributes (Malundo et al. 2001).

**FIGURE 3:**
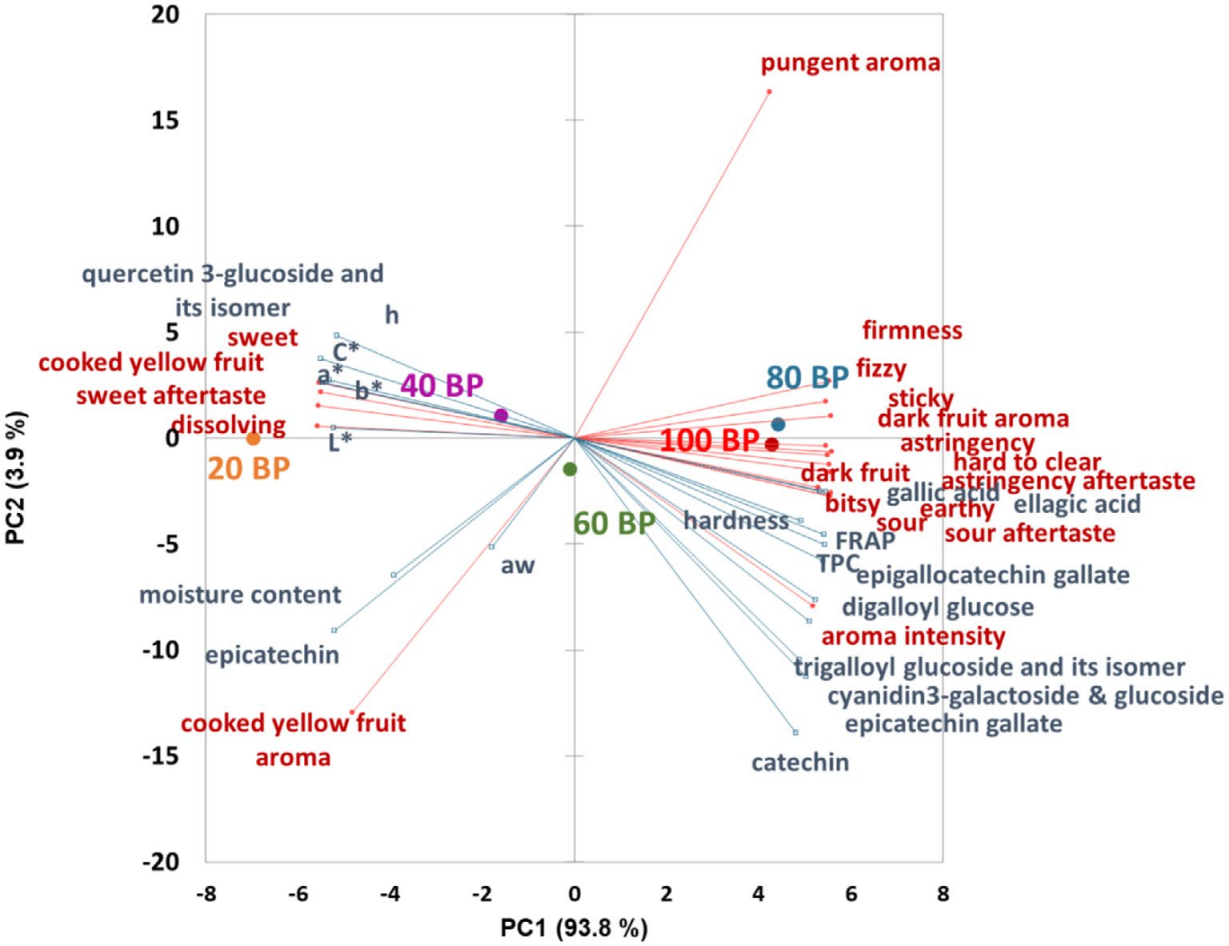
PCA biplots of tested sensory and physicochemical properties of five formulations of BP leathers. Sensory attributes are in red and supplementary instrumental variables are in blue.

The physicochemical results of five BP leathers obtained from our previous work (Chen et al. 2024) were overlaid as supplementary data on the present sensory profiling results (Figure 3), providing a more comprehensive characterization of BP leathers. The measured color parameters had a strong positive correlation, and the results projected toward 20BP, which had the highest scores of these color parameters. The instrumental hardness was projected similarly to the firmness as a result of the sensory profiling. The antioxidant capacity and most phenolic compounds were projected close to 80BP and 100BP, with sensory attributes related to BP, except for epicatechin, which was projected toward 20BP. The correlation of sensory attributes such as sour and astringent with the total phenolic content and most phenolic compounds was as expected and in agreement with previous publications (Laaksonen et al. 2013; Ramsey et al. 2021), except for epicatechin and quercetin 3‐glucoside and its isomer. Quercetin glycosides and epicatechin are the main phenolic compounds in apples and derived products (Lee et al. 2003), which can explain the positive correlation of these phenolics with 20BP (highest proportion of apple puree [80%] in the formulation). Gallic acid, ellagic acid, other phenolic compounds, and organic acids have been found to contribute to astringency and sourness in different fruits (Huang et al. 2023; Preys et al. 2006; Wu et al. 2022). Their interaction with salivary proteins, trigeminal receptors, and epithelial cells might be responsible for these effects; however, the exact mechanism of action is not clear yet (Huang and Xu 2021).

### 3.2 Preliminary Acceptability of Five BP Leathers

**FIGURE 4:**
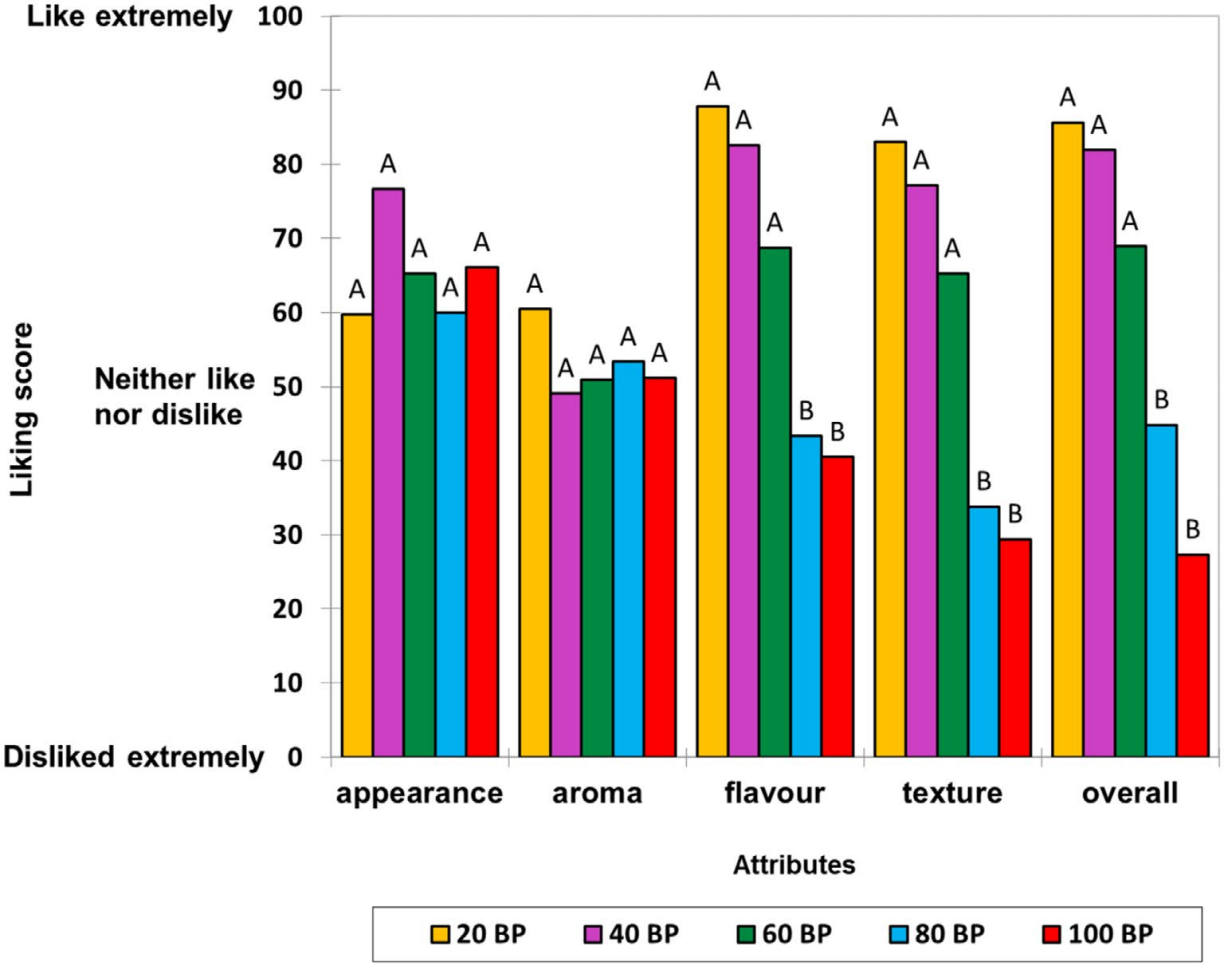
Mean acceptability of five formulations of BP leathers (*n* = 12 panelists). Data without common letters for individual attributes indicate significant differences between the samples (Tukey HSD at *p* < 0.05).

The acceptability of five BP leathers is shown in Figure 4. No significant difference was found in appearance and aroma, although 40BP and 20BP were rated on average higher on these two attributes than the rest of the samples. In terms of flavor, texture, and overall likings, 20BP, 40BP, and 60BP were rated higher (above 50) than 80BP and 100BP (below 50). This result indicated that on average, 20BP, 40BP, and 60BP were liked more than 80BP and 100BP, respectively.

When asked what the panelists liked about the appearance of the samples, their responses mainly related to the color, which was dominated by the following descriptors: translucent/matt/opaque, nice/rich/intense, and dark red/ruby/rose. There were also positive comments about the BP leathers having visible fruit pieces and a similar appearance to commercially available fruit leathers. However, a few negative comments were also given, which included visible particles and bubbles. Especially, the slight brown color of 20BP was described as pale and faded and was disliked. Regarding aroma, the reason for liking was mostly the fruity aroma, while disliking included the sweaty, savory, earthy, and metallic note. In terms of flavor, reasons for liking included sweet, sour, and fruity, while those for disliking included too sour, astringent, and bitter. Regarding texture, soft, smooth, fizzy, thin, and easy to bite and chew were listed as the main likings, while astringent, fibrous, chewy, and hard to clear were the main reasons for dislike.

With the increasing ratio of apple fruit puree in the BP leather formulations, the flavor, texture, and overall liking scores increased. Combining the results from the descriptive sensory analysis, it is apparent that sweeter samples were preferred. BPs are characterized by being sour, astringent, and fibrous (Chen et al. 2023). Royal Gala apples have a relatively high sugar content and are perceived as sweet (da Mendes Silva et al. 2021). Previous sensory studies on fruit acceptance also found that acceptance improves when sweetness is increased and sourness is reduced (van Stokkom et al. 2018). Generally, astringency tends to have a negative effect on acceptance, although its impact also depends on consumers' familiarity with the inherent characteristics of the consumed food (Dinnella et al. 2011). Earthy flavor is typically undesirable and negatively correlated with liking. However, it can also be seen as a desirable and appropriate flavor in some foods such as beetroot (Bach et al. 2015).

Besides taste, the addition of apple puree to BP leathers reduced the firmness when biting into the leather and increased the dissolvability in the mouth. Consumers' preference for softer fruit leather has also been reported (Huang and Hsieh 2005). Fruit leather with a higher ratio of apple fruit has lower bitsy sensation and is easier to clear in the mouth after consumption. These factors might explain the higher liking for 20BP, 40BP, and 60BP compared to 80BP and 100BP, respectively.

Based on the preliminary acceptability and sensory profiling results, the 40BP formulation was selected for the consumer acceptability study. Among the five formulations, 20BP, 40BP, and 60BP had relatively high liking scores. However, the sensory profile of 20BP was different from the 100BP in most attributes, resulting in a loss of the characteristic BP attributes. 40BP and 60BP had similar sensory attributes, and the 40BP formulation was selected for further study since less BP is needed, which could be an economical advantage.

### 3.3 Proximate Composition of Selected BP Leather

The proximate analysis showed that 40BP contained 23.3 ± 0.5% moisture content, 17.8 ± 1.1% dietary fiber, 2.2 ± 0.2% fat, 1.5 ± 0.1% protein, 0.720 ± 0.003% ash, and 54.5 ± 0.4% available carbohydrate. The fiber content in the 40BP leather was higher than that found in most commercial fruit leather products (less than 12%) (Sukasih and Widayanti 2022). Dietary fiber helps maintain a healthy gut including a healthy gut microbiota (Cronin et al. 2021; Makki et al. 2018), alleviates postprandial glucose levels and reduces the risk of cardiovascular diseases (Barber et al. 2020). However, less than 30% of adults and 50% of children in Australia meet the adequate intake of dietary fiber (between 14 and 30 g/day for children and adults) (Fayet‐Moore, Cassettari, et al. 2018; Fayet‐Moore, George, et al. 2018). A “cost of illness analysis” revealed that billions of dollars could be saved in healthcare expenditure and productivity losses associated with cardiovascular diseases and type 2 diabetes in Australia alone by simply increasing the cereal fiber intake (Fayet‐Moore, Cassettari, et al. 2018; Fayet‐Moore, George, et al. 2018). The increasing number of health‐conscious consumers and the awareness of the health benefits of dietary fiber have been shown to drive the demand for fiber rich foods (Kowalska et al. 2022). Fiber enrichment is among the top five food product development trends globally (Boukid et al. 2022). BP leather has the potential to serve as a valuable ingredient for enhancing the fiber content in food products.

### 3.4 BP Leather and TM Acceptability

**FIGURE 5:**
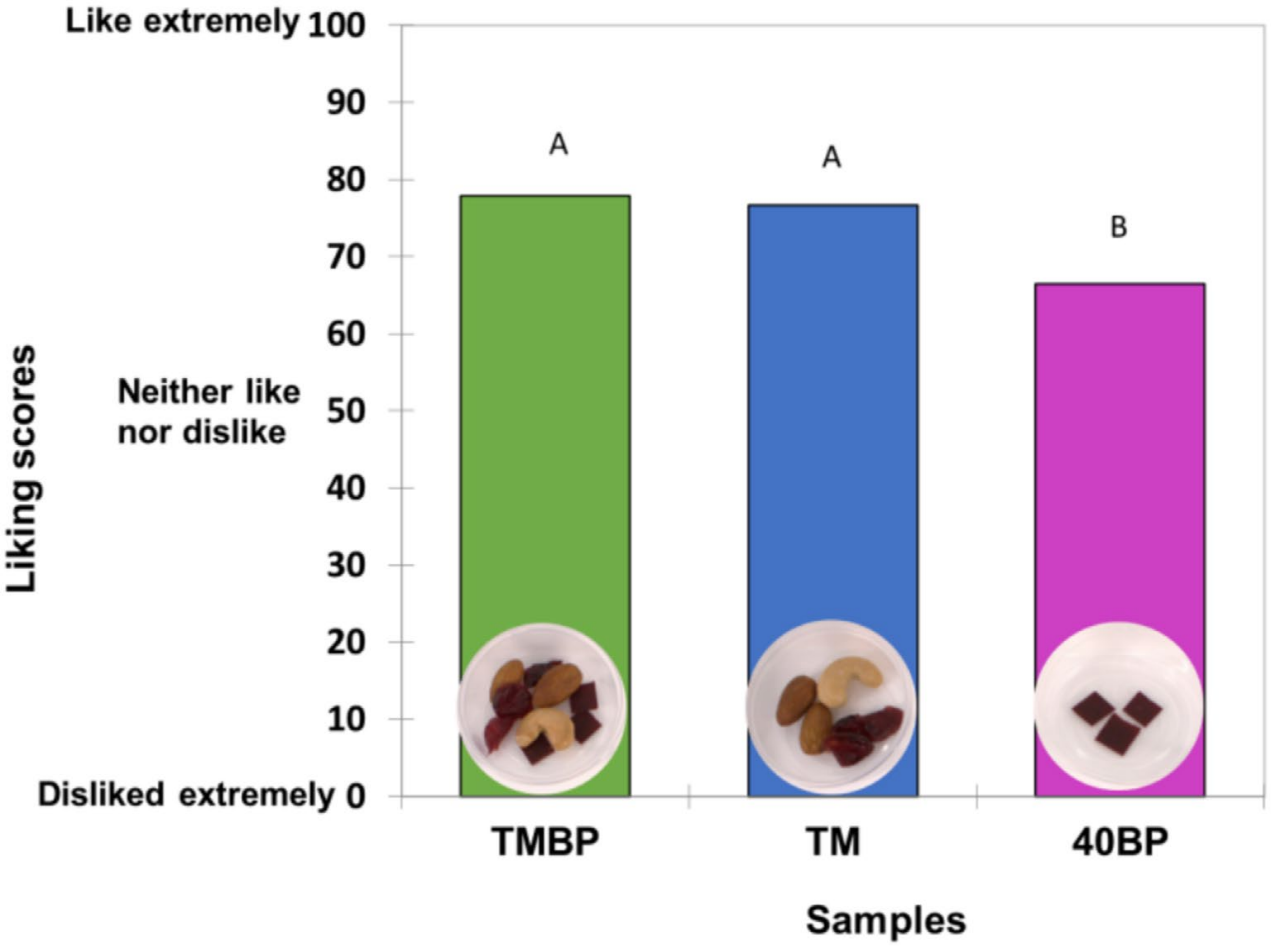
Mean sensory acceptability of fruit leather (40BP), trail mix (TM), and their combination (TMBP) (*n* = 121).

The results of the acceptability in sensory and purchase intention of 40BP, TM and TMBP are shown in Figure 5 and Table 4. The sensory acceptability results show that all three samples were liked, having acceptability scores greater than 50 out of 100. On average, the liking scores for 40BP (66 ± 16) corresponded to “like moderately” and for TM (77 ± 10) and TMBP (78 ± 11) were “like very much” on the scale. The overall liking scores of 40BP were higher (*p* < 0.05) when consumed together with the TM than when consumed on its own. Around 50% of participants were willing to purchase 40BP and that increased to around 80% for TMBP. This indicated that adding BP leather to the TM considerably increased consumers' overall liking of and purchase intention of BP leather. This corresponds to the phenomenon that a combination of foods can alter the consumer acceptability of an individual food (Nguyen and Wismer 2020; van Eck and Stieger 2020).

**TABLE 4:** Purchase acceptability of fruit leather (40BP), trail mix (TM), and their combination (TMBP) (*n* = 121).

Purchase intention (%)	40BP	TM	TMBP
1. Definitely would not purchase	5 a	1 a	1 a
2. Probably would not purchase	19 a	2 b	4 b
3. Might or might not purchase	28 a	21 a	17 a
4. Probably would purchase	33 a	42 a	40 a
5. Definitely would purchase	15 b	33 a	38 a

*Note:* Data without a common letter in each row indicate significant differences between samples for Marascuilo procedure *p* < 0.05.

The changes in sensory perception and liking of individual products when consumed together have been attributed to two major reasons, including physicochemical and cognitive interference (van Eck and Stieger 2020). The present results demonstrate the potential of BP to be processed into fruit leather and to be served in a TM. Higher acceptability scores are likely if the nutritional and health benefits of BP are provided to the participants prior to consumption. Several studies have found an improved acceptability of food products when information about their nutritional quality and potential health benefits was provided to the participants (Baker et al. 2022; Laureati et al. 2016; Verneau et al. 2019).

**FIGURE 6:**
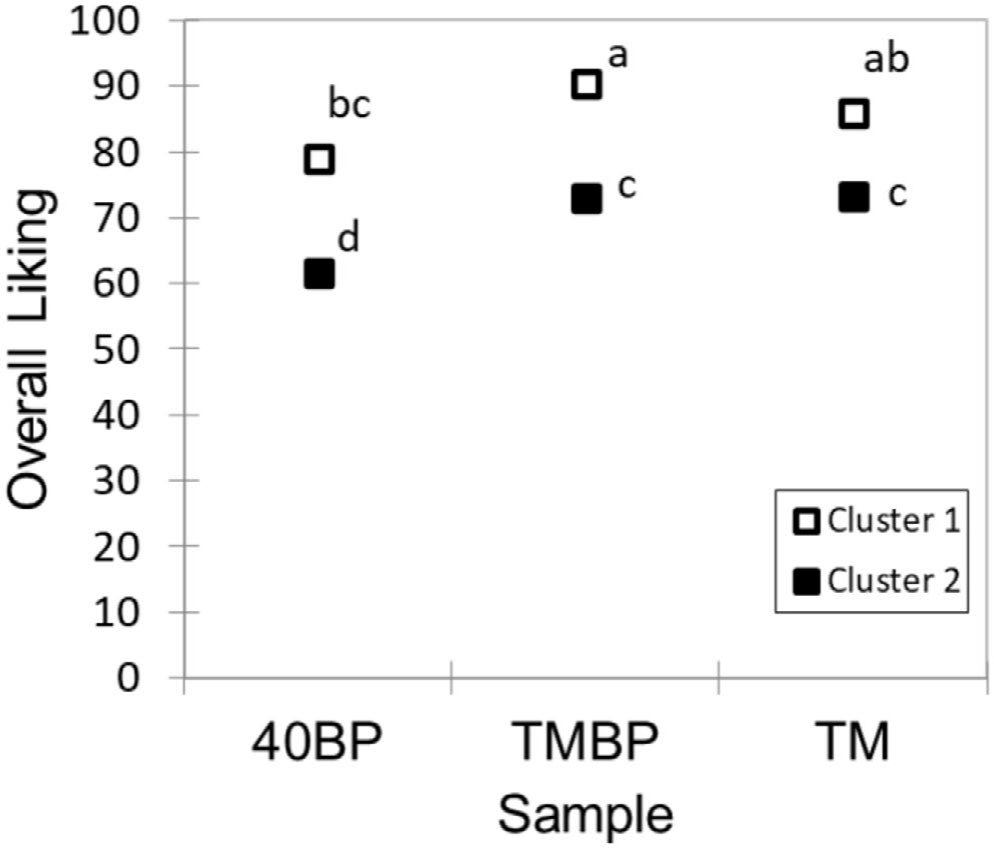
Mean overall liking of fruit leather (40BP), trail mix (TM), and their combination (TMBP) by clusters. *n* = 33 for cluster 1 and *n* = 88 for cluster 2. Data without a common letter indicate significant differences between samples (Tukey HSD at *p* < 0.05).

**FIGURE 7:**
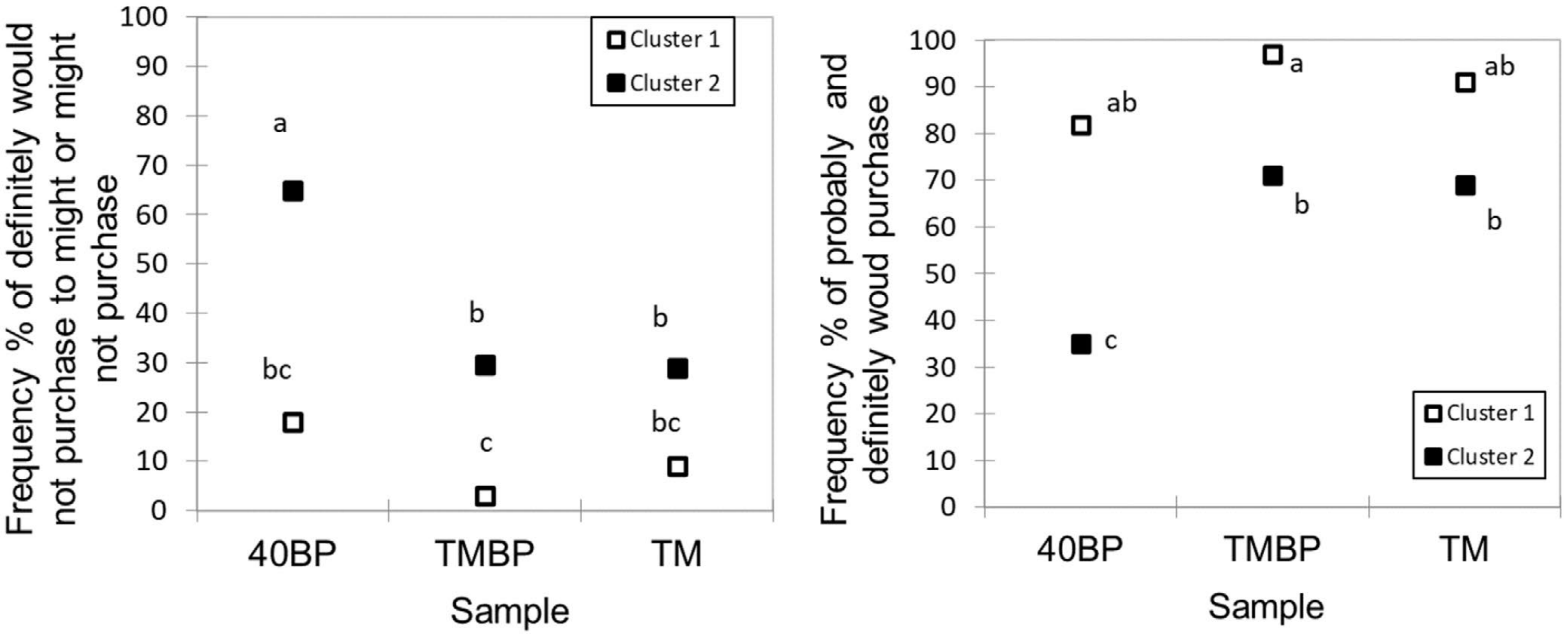
Purchase intention of fruit leather (40BP), trail mix (TM), and their combination (TMBP) by clusters *n* = 33 for cluster 1 and *n* = 88 for cluster 2. Data without a common letter indicate significant differences between samples for Marascuilo procedure at *p* < 0.05.

The AHC analysis of liking score data classified participants into two clusters (*n* = 33 for Cluster 1 and *n* = 88 for cluster 2). There was heterogeneity in acceptability among participants. On average, cluster 1 had a significantly (*p* < 0.05) higher liking for all three samples than cluster 2 (Figure 6). For both clusters, 40BP was less liked when consumed alone than in combination with TM. Particularly, cluster 2 participants rated 40BP significantly (*p* < 0.05) lower than TM. Also, cluster 2 participants had a similar liking for TM and TMBP, indicating their weaker hedonic discriminability of these two samples. In terms of purchase intention (Figure 7), cluster 1 showed a higher intention/willingness of the participants to purchase 40BP and TMBP than cluster 2 (27% vs. 10% for 40BP and 76% vs. 24% for TMBP, respectively). However, being undecided and not willing to purchase 40BP and TMBP was lower in cluster 1 than in cluster 2 (18% vs. 65% for 40BP and 3% vs. 30% for TMBP, respectively). Both clusters had a similar purchase intention for TM.

While sensory acceptability drives consumer choices, other factors including individual differences can also play a role (Resano et al. 2009). This study collected participant data including gender, age, consumption frequency, and consumption habits, prior knowledge of BP, and consumption attitudes toward Australian native fruits (Table 5). Most of the participants interviewed were female (61%) and aged between 25 and 44 years (65%). At the highest consumption frequency (weekly), consumers reported higher consumption for fruit and nut TMs (39%) than fruit leather (14%). This was in support of previous findings that dried fruits are more often consumed with other ingredients/foods than alone (Jesionskowska et al. 2008). In terms of prior knowledge, the results showed that BP is a lesser‐known native fruit, with 55% of the participants never having heard of it and only 7% having purchased and tasted it before. In contrast, around 95% of the participants were willing to eat Australian native fruits. This highlights the need to promote individual Australian native fruits such as BPs. While there is no guaranteed pathway for the successful commercialization of new food products, increasing public awareness of the food and its products will most likely increase their acceptance (Verbeke 2005) and thus be crucial in driving market demand and investment (Schultz et al. 2009).

**TABLE 5:** Participant characteristics (*n* = 121).

Characteristic	Category	Total %	Cluster 1 (*n* = 33) %	Cluster 2 (*n* = 88) %
Gender	Female	61	66	59
	Male	39	33	41
	Other	0	0	0
Age	65 and over	2	0	2
	55–64	15	21	13
	45–54	12	21	9
	35–44	25	22	26
	25–34	40	30	43
	18–24	7	6	7
Fruit and nut trail mixes consumption frequency	Rarely or never	10	3 b	12 a
	A couple of times per year	18	9 a	22 a
	At least once per month	33	30 a	34 a
	At least once per week	39	58 a	32 b
Fruit and nut trail mixes consumption habit	Ate fruit and nut together	53	67	47
	Ate fruit and nut separately	47	33	52
Fruit leather consumption frequency	Rarely or never	47	45	48
	A couple of times per year	25	24	25
	At least once per month	14	6	17
	At least once per week	14	24	10
Prior knowledge of Burdekin plum	Purchased and tasted it (raw or processed)	7	9	6
	Tasted it (raw or processed) but not purchased	12	12	12
	Heard of it but not tasted it (raw or processed)	26	27	26
	Not heard of it (raw or processed)	55	52	56
Willingness to eat Australian native fruits	Definitely willing	70	91 a	63 b
	Probably willing	25	9 b	31 a
	Might or might not willing	4	0 b	6 a
	Probably not willing	1	0	1
	Definitely not willing	0	0	0

*Note:* Data without a common letter in the same row indicate significant differences for Marascuilo procedure at *p* < 0.05.

The chi‐square analysis revealed the association between the clusters and two participant characteristics: consumption frequency of fruit and nut TMs and consumption attitudes toward Australian native fruits. The number of participants who frequently consumed fruit and nut TMs was higher in cluster 1 than in cluster 2 (58% vs. 32%) and also the willingness to eat Australian native fruits (91% vs. 63%). These findings support previous studies reporting that an increase in consumption frequency, familiarity, and positive attitudes toward specific foods can positively affect the liking of these foods (Embling et al. 2022; Nacef et al. 2019). The mere exposure effect is a potential explanation for the increase in liking, which suggests that repeated exposure can increase familiarity and reduce negative perception (Garcia‐Marques et al. 2016; Montoya et al. 2017).

## 4 Conclusion

The present study contributed to our understanding of the sensory properties and consumer preferences of BP leathers and TM. The descriptive sensory analysis showed that 80BP and 100 BP were similar in all 23 measured sensory attributes, whereas the 20BP (lowest content of BP) was significantly different from the 80BP and 100BP in 19 attributes. PCA revealed that most attributes were highly correlated. Only the 40BP formulation was selected, based on the preliminary acceptability evaluation, and subjected to the sensory acceptability test together with the commercial TM. This was because the study aimed to select one fruit leather formulation for consumer testing, and such tests typically involve a large number of participants. It is generally recommended that fewer than five samples be included in a single session to minimize sensory fatigue (Stone et al. 2021). The acceptability results demonstrated that TM is an appropriate companion for BP leather and adding BP leather to the TM can improve the acceptability of BP leather. However, the participants were recruited from a conference setting, which may limit the generalizability of the findings to a broader consumer population. Furthermore, 40BP was also found to be rich in dietary fiber. Taking all results into account, the present study clearly demonstrated the potential of BP leather and its TM as feasible food applications of BP. This offers valuable insights into enhancing the overall appeal of other underutilized native fruits.

## Author Contributions

**Gengning Chen:** conceptualization (lead), data curation (lead), formal analysis (lead), investigation (lead), methodology (equal), project administration (lead), resources (equal), software (lead), validation (lead), visualization (lead), writing – original draft (lead), writing – review and editing (lead). **Sandra Milena Olarte Mantilla:** conceptualization (supporting), data curation (supporting), formal analysis (supporting), investigation (supporting), methodology (equal), resources (supporting), software (supporting), supervision (equal), validation (supporting), visualization (supporting), writing – review and editing (supporting). **Michael E. Netzel:** funding acquisition (supporting), supervision (supporting), writing – review and editing (supporting). **Daniel Cozzolino:** funding acquisition (supporting), supervision (supporting), writing – review and editing (supporting). **Yasmina Sultanbawa:** conceptualization (supporting), funding acquisition (lead), resources (equal), supervision (equal), writing – review and editing (supporting).

## Ethics Statement

Ethical approval for the involvement of human subjects in this study was granted by the University of Queensland Human Research Ethics Committee A (approval number 2019002607). Informed consent was obtained from all participants.

## Conflicts of Interest

The authors declare no conflicts of interest.

## Supporting information


Table S1.
Table S2.
Table S3.

## Data Availability

The data that support the findings of this study are available within the article and/or its supplementary material.
