# Supplementary material for: Sensory Profiling of Burdekin Plum Leathers and Consumer Acceptability of Its Combination With Trail Mix
Source: Food Sci Nutr. 2025 May 12;13(5):e70277. doi: 10.1002/fsn3.70277 (PMC12066820; doi:10.1002/fsn3.70277)
Supplement: Supplementary file 1 — Table S1. Table S2. Table S3. [file FSN3-13-e70277-s001.docx]

**Sensory profiling of Burdekin plum leathers and consumer acceptability of its combination with trail mix**

**Supplemental Information**

Table S 1. Panellist' performance score based on ANOVA model (n=5 samples x 3 replicates x 12 panellists)

| Panellist | 1 | 2 | 3 | 4 | 5 | 6 | 7 | 8 | 9 | 10 | 11 | 12 |
| --- | --- | --- | --- | --- | --- | --- | --- | --- | --- | --- | --- | --- |
| Discrimination | 8 | 12 | 12 | 15 | 14 | 13 | 15 | 15 | 16 | 13 | 16 | 14 |
| Repeatability | 17 | 15 | 16 | 17 | 14 | 16 | 17 | 17 | 16 | 18 | 14 | 9 |
| Total | 25 | 27 | 28 | 32 | 28 | 29 | 32 | 32 | 32 | 31 | 30 | 23 |

The score represents the number of attributes being discriminated or having repeatability by panellists

Table S 2 Summary of descriptive analysis scores (n=5 samples x 3 replicates x 12 panellists)

| Sensory attribute | Mean | SD | CV% | SEM | Minimum | Maximum |
| --- | --- | --- | --- | --- | --- | --- |
| **Aroma (none – high, 0-100)** |  |  |  |  |  |  |
| *aroma intensity* | 57 | 23 | 40 | 1.8 | 42 | 72 |
| *dark fruit* | 53 | 25 | 47 | 1.9 | 26 | 72 |
| *cooked yellow fruit* | 30 | 27 | 89 | 2.1 | 3 | 60 |
| *green* | 31 | 29 | 95 | 2.3 | 0 | 66 |
| *savoury* | 27 | 29 | 107 | 2.2 | 0 | 63 |
| *sweet spice* | 15 | 20 | 137 | 1.6 | 1 | 39 |
| *pungent* | 29 | 29 | 100 | 2.3 | 3 | 54 |
| **Flavour (none – high, 0-100)** |  |  |  |  |  |  |
| *sour* | 55 | 32 | 57 | 2.5 | 36 | 68 |
| *sweet* | 40 | 33 | 81 | 2.5 | 23 | 53 |
| *dark fruit* | 61 | 27 | 44 | 2.1 | 32 | 73 |
| *cooked yellow fruit* | 39 | 33 | 83 | 2.5 | 24 | 76 |
| *earthy* | 30 | 31 | 104 | 2.4 | 0 | 68 |
| **Texture (none – high, 0-100)** |  |  |  |  |  |  |
| *firmness* | 55 | 33 | 61 | 2.6 | 45 | 63 |
| *dissolving* | 44 | 34 | 77 | 2.6 | 35 | 56 |
| *bitsy* | 54 | 31 | 58 | 2.4 | 28 | 64 |
| *sticky* | 42 | 29 | 70 | 2.3 | 15 | 67 |
| *fizzy* | 48 | 31 | 64 | 2.4 | 33 | 68 |
| *astringency* | 54 | 31 | 59 | 2.4 | 35 | 69 |
| **Taste and mouthfeel after swallowing** |  |  |  |  |  |  |
| *sour* | 50 | 30 | 60 | 2.3 | 25 | 61 |
| *sweet* | 35 | 30 | 84 | 2.3 | 16 | 50 |
| *fruit length* | 56 | 25 | 45 | 1.9 | 28 | 78 |
| *astringency* | 48 | 32 | 66 | 2.5 | 16 | 65 |
| *hard to clear* | 46 | 29 | 64 | 2.3 | 19 | 58 |

Table S 3. F ratios and significance for samples, panel and replicate (n=5 samples x 11 panellists x 3 replicates)

| Sensory attribute | Sample | Panel | Replicate |
| --- | --- | --- | --- |
| aroma intensity | 5.3** | 3.3** | 0.97 |
| dark fruit aroma | 2.7* | 5.2*** | 1.17 |
| cooked yellow fruit aroma | 3.6* | 11.7*** | 0.1 |
| green aroma | 0.9 | 14*** | 4.15* |
| savoury aroma | 1.1 | 15.6*** | 1.05 |
| sweet spice aroma | 0.7 | 5.5*** | 1.56 |
| pungent aroma | 5.5** | 8.2*** | 0.67 |
| sour | 77.5*** | 4.2*** | 0.39 |
| sweet | 86.8*** | 3.9*** | 0.69 |
| dark fruit | 16.1*** | 4.3*** | 3.11* |
| cooked yellow fruit | 35.6*** | 5.7*** | 0.72 |
| earthy | 11*** | 8.6*** | 0.21 |
| firmness | 152.7*** | 1.9** | 2.59 |
| dissolving | 70.9*** | 1.2 | 1.13 |
| bitsy | 36.8*** | 2.8** | 0.02 |
| sticky | 3.5* | 5.1*** | 0.81 |
| fizzy | 60.2*** | 5*** | 1.11 |
| astringency | 50.1*** | 4.6*** | 0.83 |
| sour aftertaste | 82.2*** | 7.4*** | 0.06 |
| sweet aftertaste | 52.2*** | 3.4** | 0.45 |
| fruit length | 0.4 | 2.9** | 0.08 |
| astringency aftertaste | 52*** | 8.2*** | 1.52 |
| hard to clear | 28.9*** | 2.5* | 0.39 |

Significant F-ratios are indicated by * (p < 0.05), ** (p < 0.01), *** (p < 0.001)
